# Integration of hydrogels in microfabrication processes for bioelectronic medicine: Progress and outlook

**DOI:** 10.3389/fbioe.2023.1150147

**Published:** 2023-03-24

**Authors:** Saloua Saghir, Kristin Imenes, Giuseppe Schiavone

**Affiliations:** Department of Microsystems, Faculty of Technology, Natural Sciences and Maritime Sciences, University of South-Eastern Norway, Horten, Norway

**Keywords:** hydrogels, bioelectronic interfaces, microfabrication processes, implantable medical devices, biomaterials, soft bioelectronics, medical device encapsulation, biomimetic coatings

## Abstract

Recent research aiming at the development of electroceuticals for the treatment of medical conditions such as degenerative diseases, cardiac arrhythmia and chronic pain, has given rise to microfabricated implanted bioelectronic devices capable of interacting with host biological tissues in synergistic modalities. Owing to their multimodal affinity to biological tissues, hydrogels have emerged as promising interface materials for bioelectronic devices. Here, we review the state-of-the-art and forefront in the techniques used by research groups for the integration of hydrogels into the microfabrication processes of bioelectronic devices, and present the manufacturability challenges to unlock their further clinical deployment.

## 1 Introduction

Since inception of the first-in-human implanted device in the form of an artificial pacemaker in 1932 (Aquilina, 2006), integrated electronics interfacing directly with biological tissue have widely grown in interest. Conditions such as bladder dysfunction (Clausen et al., 2018), chronic pain (Finch et al., 2019), spinal cord injury (Wagner et al., 2018), neurodegenerative diseases (Khedr et al., 2019) and heart failure (Nguyen et al., 2016; Jorbendaze et al., 2022) are increasingly treated with medical devices establishing electrical interactions with the targeted organs or the nervous structures governing their functions. Bioelectronic medical devices are implanted into the biological host to provide diagnostic and/or therapeutic capabilities, most commonly in the form of signal recording and electrical stimulation. Recent research bridging the fields of medicine and micro- and nanotechnology aims to unlock the fabrication of bioelectronic devices that leverage microfabrication techniques and their inherent advantages of miniaturization and design flexibility for personalization (Vomero and Schiavone, 2021). Historically, the first micrometre-sized bioelectronic devices, embodied as recording neural electrode arrays, spurred from the advances in semiconductor manufacturing of the 1970s (Scholten and Meng, 2015). Despite the maturity and reliability of silicon technology, however, the rigidity of the associated substrate, conductor and insulating materials, quantified by Young’s moduli *E* in the 100 GPa range, together with planar form factors, introduces a mechanical mismatch to soft biological tissues (*E* in the kPa range), hindering seamless device biointegration (Lacour et al., 2016).

## 2 Biointerface matching: Mechanical, chemical and electrical

Mechanical mismatch results from a combination of material properties and device geometry. In the case of surface-deployed devices (membrane-like), their stiffness can be estimated by the flexural rigidity, which is proportional to the materials’ Young’s moduli and the cubic power of the thickness. A possible solution to lower the mechanical mismatch is therefore to reduce the substrate thickness. This strategy has materialized in ultra-thin (<10 µm) bioelectronic interfaces using GPa-range polymers such as SU-8, Polyimide and Parylene-C as substrate material (Cho et al., 2008; Luan et al., 2017; Vomero et al., 2022). While effectively lowering the flexural rigidity, ultra-thin form factors introduce significant challenges in device handling, requiring complex *ad hoc* tools to enable surgical manipulation (Luan et al., 2017; Vomero et al., 2020), and possible limitations to area coverage (less than few cm^2^). An alternative strategy to lower the mechanical mismatch is to use softer materials (*E* < 1 MPa) for the structural elements of the devices. For a given flexural rigidity value, using low modulus materials enables larger thicknesses and therefore easier handling (Fallegger et al., 2021). While initial steps in this direction have been explored in recent years with elastomer-based bioelectronic interfaces (Lee K. Y. et al., 2020; Renz et al., 2020; Schiavone et al., 2020a), further advances may be achieved by using even softer materials. Hydrogels (E < 100 kPa) for instance, can be engineered to present mechanical, chemical and electrical properties enabling a more comprehensive matching at the device-tissue interface.

Hydrogels are defined as cross-linked polymeric networks capable of absorbing large quantity of water (up to 90% in weight) without dissolving (Li et al., 2015). Hydrophilic functional groups (e.g., COOH, NH_2_, OH) bonded to the polymer introduce affinity with water. The cross-linking between the polymer macromolecules provides the resistance to dissolution and the ability to maintain a 3D structure in the swollen state (Bashir et al., 2020). In addition to the mechanical advantages, the nature of such materials provides affinity towards the chemical properties of biological tissues (Yuk et al., 2020), characterized by high water-to-weight ratios of approximately 70% for muscles and skin, 75% for the heart, 80% for the lungs and 85% for the brain (Yuk et al., 2022). The immune system recognizes and targets hydrophobic regions of biomolecules as a universal molecular pattern associated with damage (Seong and Matzinger, 2004; Roh and Sohn, 2018). Although non-cytotoxic, the hydrophobic nature of most bioelectronic implants is thought to trigger proinflammatory protein responses (Scholten and Meng, 2015; Mariani et al., 2019), which in turn activate the immune cells responsible for the scar formation around the implanted device (Franz et al., 2011). Over long implantation periods, this phenomenon can lead to the formation of a fibrotic tissue encapsulation around the device (Prodanov and Delbeke, 2016), compromising its functionality (Williams, 2008) and potentially damaging both tissue (Biran et al., 2005) and device (Song et al., 2022). With their ability to absorb water, hydrogels are intrinsically hydrophilic. Used at the biological interface, they limit the adsorption of proinflammatory proteins by inhibiting their hydrophobic domain to attach to the surface of the device (Wellman and Kozai, 2017). At the current preclinical state of research, implants using hydrogels as interface have been shown to benefit from decreased foreign body reaction (Zhang et al., 2013; Yan et al., 2019; Gori et al., 2022).

**FIGURE 1:**
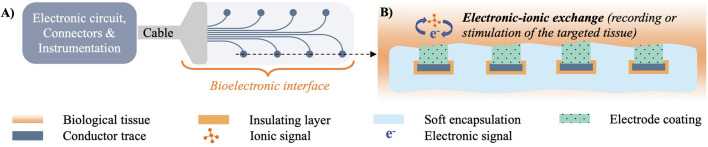
**(A)** Top view and **(B)** Cross section of an implanted bioelectronic device.

For a bioelectronic interface to electrically interact and relay information to and from biological tissue, several components are required, as shown in Figure 1. Biological tissue generates biopotentials through ionic interactions in the vicinity of a working electrode, which can use different charge transduction mechanisms to convert them into a measurable signal (Schiavone et al., 2021). Electrodes transmit such signals through embedded conductors to the electronic circuitry (e.g., solid state amplifiers, logic, power supply) that processes and relays them to the user instrumentation *via* cables, connectors and/or telemetry modules. Similarly for electrical stimulation, pulses of current are relayed through electronic circuitry to the implanted working electrode, which injects the charge to the ionic carriers in the tissue. The performance of bioelectrodes is typically measured by their electrochemical impedance and charge transduction properties, that reflect how efficiently the interface mediates the transmission of signals of different waveform, frequency and amplitude between the electronic circuitry and the ionic medium (Schiavone et al., 2020b). Although metals offer high electrical conductivity, this property *per se* does not necessarily guarantee efficient charge transduction (Seyedkhani et al., 2022), and extensive research has focused on developing optimal interface materials mediating electronic-ionic signal transmission. For instance, it has been demonstrated that metal electrodes coated with organic conductors can outperform uncoated controls (Khodagholy et al., 2011; Venkatraman et al., 2011). Materials offering mixed electronic and ionic electrical conduction exhibit in particular high charge transduction properties (Cogan, 2008). Adding to the mechanical and chemical matching advantages described in the previous paragraphs, hydrogels can be engineered to embed mixed-conduction polymers (typified by PEDOT:PSS), and therefore offer efficient ionic-electronic signal transduction at the interface with biological tissue (Ido et al., 2012; Kleber et al., 2017).

## 3 Classification, engineering and functionalization of hydrogels

Numerous classifications have been proposed for hydrogels according to different categories of material properties. Table 1 provides a comprehensive list of the possible classification criteria found in the literature, accompanied by corresponding examples of published research.

**TABLE 1:** Classification of hydrogels according to different categories of material properties.

Classification criteria	Subgroup	Description	Example
**Origin of the polymer**	Natural	• Inherent biocompatibility (Kaczmarek et al., 2020)	• Protein: Collagen, Gelatin, Silk
		• Cell growth promotion, attachment and differentiation	• Polysaccharide: Alginate, HA, Chitosan
		• Overall better affinity with biological tissues	
	Synthetic	• Stable mechanical properties	• Vinyl: PAA, PVA, PHEMA
		• Ease to process	• Polyester: PCL, PGA
		• Wide range of customization	• PEG
		• Lower affinity for the cellular environment	
	Hybrid	Combination of natural and synthetic polymers to merge cellular affinity with enhanced mechanical properties	• PVA-Alginate
			• PEG-Silk Fibroin
			• PHEMA-HA
**Composition of the hydrogel matrix**	Homopolymer	The hydrogel network is composed of a single monomer species (building block repeating itself to form the polymeric macromolecule)	• PHEMA
			• PVA
			• Chitosan
	Copolymer	Combination of two or more monomeric species where at least one is hydrophilic	• PACP-PVA (Tao et al., 2021)
			• PBMA-PMAA-PBMA (Zhang et al., 2019)
	Multipolymer or IPN	Entanglement of two distinct polymeric networks. One of the polymer is synthesized in the immediate presence of the other (Sperling, 2012) (Raina et al., 2020)	• PAA-PEDOT (4) (Fu et al., 2021)
			• GelMA-PEDOT (Bansal et al., 2022)
**Cross-linking process**	Chemical	Permanent covalent bonds between the polymer chains	• Collagen
	Physical	• Hydrogen bonds	• PVA-Chitosan
		• Hydrophobic interactions	• PVA-Gelatin
		• Ionic interactions	
		• Physical entanglement	
**Responsiveness to stimuli**	Chemical	• pH	• AG-GMA (Reis et al., 2006)
		• Glucose	• PEG (Zhang et al., 2017)
		• Oxidation	• Cellulose (Liu et al., 2017)
	Biochemical	• Antigens	• PAA-anti-HBc (Lim et al., 2020)
		• Enzymes	• PEG-LOx (Tirella et al., 2020)
		• Ligands	• PEG-PDCA (Ahmadi et al., 2022)
	Physical	• Temperature	• PNH (Zhao et al., 2009)
		• Pressure	• Agar-PAAN (Wang et al., 2020)
		• Light	• Dextran- *trans* azobenzene and cyclodextrin (Peng et al., 2010)
		• Magnetic fields	• Gelatin-CNC- Fe^2+^/Fe^3+^ (Araújo-Custódio et al., 2019)
		• Electric fields	• Alginate- Fe^3+^ (Ambrožič and Plazl, 2021)
**Resorbability**	Biodegradable	Hydrogels that are decomposed by living organisms	• Natural hydrogels
			• PCL, PEG
	Non-biodegradable	Hydrogels that cannot be decomposed by living organisms	• PAA-Cellulose (Loh)
			• PVA (Bichara et al., 2010)
	Dissolution	Chemical process in which the hydrogel solute is dissolved in a solvent	• PMEP-DMA (Tee et al., 2020)
			• SVA-PEG-SVA (Konieczynska and Grinstaff, 2017)
	Hydrolysis	Chemical reaction in which water molecules react with functional groups in the polymer chain resulting in the dissolution of the hydrogel	• PEG-VS (Zustiak and Leach, 2010)
			• PEG-PAMAM (Buwalda et al., 2019)
**Charge carrier**	Ionic	• Anionic	• HA, pectin, dextran
		• Cationic	• Chitosan
	Zwitterionic	Anionic and cationic groups in each repeating structural block	• PEG, polysulfobetaine, polycarboxybetaine, polyectoine
	Ampholytic	Contains both acidic and basic functional groups to carry on the electrical charges	• Collagen, gelatin, chitin, fibrin

One of the most desirable features of hydrogels is the ability to modify and fine-tune their properties to render them adapted to a broad range of applications. They can be customized to achieve better affinity with the cellular environment, tailored to mimic the water content of biological tissue, and synthesized with precise mechanical properties such as higher elasticity and conformability. In the following section, we present techniques that have been used to engineer hydrogel materials to optimize their function in bioelectronic devices.

### 3.1 Mechanical properties and porosity

The elastic modulus of hydrogels is proportional to the density of crosslinks between the polymer chains, the crosslink length and the molecular weight of the precursor (Li et al., 2018). One of the properties that limits hydrogel use, particularly in medical applications, is their brittle nature. Several strategies have been proposed to overcome this challenge, such as using IPN matrix gels (Dragan, 2014), loading the hydrogel with nanoparticles (Chen et al., 2021) or nanofibers such as silk fibroin (Cui et al., 2021), as well as chitin (Ge et al., 2018; Suo et al., 2018). Plasticizers such as lauric acid, glycerol, sorbitol and PEG constitute an alternative option to increase flexibility and tensile strength especially in natural hydrogels without compromising cell viability (Reis et al., 2006; Snejdrova and Dittrich, 2012; Sun et al., 2018; Tarique et al., 2021).

In biointerfaces, the pore size and distribution within the 3D hydrogel matrix play an important role in cell growth (Přádný et al., 2014), with large pores (20–80 µm) enabling cell proliferation and smaller pores (1 µm) allowing for nutrient and oxygen supply (Janoušková et al., 2019). For electrochemical electrode interfaces, porosity increases the electroactive surface area of a working electrode (Zhu and Zhao, 2017) allowing for more efficient charge injection or higher quality signal recording (Cogan, 2008). Porosity can be engineered by lyophilization, a process in which the hydrogel is quickly frozen to induce a phase separation between the polymeric network and the solvent. The latter is then removed by sublimation, leaving cavities in the parts it previously occupied. Another method is gas foaming, where a foaming agent chemically reacts with the precursor hydrogel solution to create bubbles (e.g., CO_2_ formed in acidic environment). Electrospinning can also be used to fabricate fibrous and porous hydrogel scaffolds. The fibres are created by applying an external voltage to the precursor polymeric solution, which is then ejected through a spinneret. Part of the solvent volatilizes and the filaments are collected on a collector plate (Annabi et al., 2010).

### 3.2 Electrical conductivity

Electrical conductivity can be mediated either ionically or electronically. Owing to their ability to absorb biological fluids where ionic interactions are predominant, hydrogels can indirectly act as ionic conductors. In this case, the electrical conductivity of ionic hydrogels (0.1–10 S/m) (Yang and Suo, 2018) remains comparable to that of biological tissue (0.03–1.6 S/m) (Martinsen et al., 2008). It is possible to further increase ionic conductivity by doping the hydrogel with salts such as NaCl, LiCl, FeCl_3_, KCl or CaCl_2_, but only to a certain extent, since excessive concentrations can cause tissue damage (Peng et al., 2020). Another option is to use conductive polymers such as PPy, PEDOT, PANi. The aromatic groups in the polymeric chains contain π-conjugations (alternating single and double covalent bonds) with free electrons, conferring electronic conductivity (10^–3^–10^5^ S/cm) (Nezakati et al., 2018; Gao et al., 2022).

More recently, doping of hydrogels with conductive nanomaterials has gained interest as a method to achieve higher conductivity ranges. As notable examples, hydrogels doped with silver particles have displayed conductivities in the range of 1.36–374 S/cm (Devaki et al., 2014; Ohm et al., 2021), while doping with graphene or carbon nanotubes has enabled conductivities of 4 × 10^−5^–4.2 × 10^−3^ S/cm (Zhou et al., 2018; Park et al., 2019) and 0.01–10 S/cm (Cho and Borgens, 2010; Liu et al., 2014), respectively.

When discussing conductivity in biointerfaces, it is worth noting that bioelectronic devices require spatially confined electrically conductive regions in contact with tissue (i.e., electrode contacts), while the remaining device surface must be electrically insulated. This ensures that electrical interfacing is established at precisely defined locations only. As hydrogels absorb the surrounding ionically conductive fluids, they cannot be used as insulation for electrodes and conductors, and careful device design integrating suitable barrier layers is required to ensure correct functionality.

### 3.3 Adhesion

When hydrogels are used as interface material between a bioelectronic device and biological tissue, controlling the adhesion to both parts is crucial. The abundant functional groups present on biological tissues such as hydroxyl, carboxylic acid, thiol and amino groups can be leveraged to anchor the hydrogel *via* chemical or physical bonds (Yang et al., 2022). Physical binding results mainly from electrostatic, Van der Walls and hydrogen bonds, and is by nature weaker than covalent bonding. To promote adhesion, hydrogels can be functionalized with chemical moieties capable of binding to the functional groups naturally present in tissues. Common chemical reactions to form covalent bonds to tissue are Michaelson’s addition and Schiff’s base reaction (Cong and Fu, 2022). Additionally, several nature-inspired approaches have also been demonstrated. Mimicking mussels’ ability to adhere to surfaces in sea water, polydopamine has been introduced as a plaster to promote adhesion to wet surfaces (Chalmers et al., 2020). The catechol, imine and amine groups present in polydopamine allows adhesion on different surfaces (e.g., PDMS, paper, PI, glass, metals) through π-π stacking, hydrogen and covalent bonding (Ryu et al., 2018). A third option is mechanical intermeshing, achievable by surface roughness or microstructured patterns (Fan and Gong, 2021). Notable examples are reversible adhesion by octopus-inspired microstructures (Lee et al., 2016) and hexagonal mesh grids of clingfish (Rao et al., 2018).

### 3.4 Self-healing

In analogy to the resilience of biological tissues, self-healing hydrogels have been introduced with the ability of restoring their electrical and mechanical properties after rupture (Lei et al., 2017; Deng et al., 2018; Robby et al., 2019; Talebian et al., 2019; Ge et al., 2020; Su et al., 2021). This recovery property originates from reversible bonds present within the material architecture (Taylor and in het Panhuis, 2016), and more specifically owing to the intrinsic ability of chemical molecules to recreate a bond after rupture. Healing mechanisms have been reported involving both covalent and non-covalent, ionic or hydrogen, bonds. For example, hydrogen or ionic bonds can be broken and re-established between, respectively, two hydroxyl groups (OH-OH), or carboxyl groups (COOH) and Fe^3+^ (Devi V. K. et al., 2021). Reversible covalent bond break and formation has equally been reported between disulfide (S-S) and imine bonds (C=N) (Talebian et al., 2019).

### 3.5 Loading with pharmacological agents

In pharmacological applications, hydrogels have been extensively employed to facilitate the release of concentrated drugs or chemical molecules over a prolonged period of time through diffusion, swelling or environmental stimuli. The problems of systemic toxicity and repeated administration that conventional drug carriers might cause can be avoided by hydrogel-mediated release (Narayanaswamy and Torchilin, 2019). Specifically for implanted bioelectronic devices, hydrogel loading with drugs to inhibit inflammatory response in the surrounding tissue has been proposed (Nguyen et al., 2022). Growth factors are another type of bioactive molecules that can be incorporated into hydrogel matrices. They are crucial factors in tissue repair and regeneration, however biostability and yield challenges related to their rapid degradation before reaching the target are yet to be fully solved (Tayalia and Mooney, 2009). The use of electrical stimulation to facilitate the release of growth factor molecules (e.g., neurotrophins, myostatin, thrombopoietin) embedded in conductive hydrogel carriers in a controlled manner has shown great potential for cell adhesion, proliferation and differentiation (Liu et al., 2021; Cheah et al., 2023). The porous structure of conductive hydrogels offers an additional advantage compared to the polymer counterparts, as larger amounts of bioactive molecules can be stored within the matrix and released for a prolonged period of time (Caballero Aguilar et al., 2019).

### 3.6 Degradability

Degradability is an essential parameter for implantable devices. Depending on the therapeutic timeframes, strategies towards long- or short-term degradation can be employed. Degradability can be tailored by using intrinsically biodegradable material (e.g., HA, collagen) or by integrating molecules or degradable polymeric segments into the hydrogel matrix. Functional groups such as esters, anhydrides and thioesters are subject to hydrolysis. Incorporated to the polymeric chains, they can react with water and/or enzymes, leading to the dissolution of the hydrogel (Ozcelik, 2016). Copolymeric hydrogels containing alternatively synthetic polymeric sequences and peptide or protein units are commonly used to fabricate biodegradable composites (Kopeček and Yang, 2007; Patterson et al., 2010). The use of low crosslinking degree and low molecular weight crosslinkers is another way to promote degradation (Kong et al., 2004). In cases where degradation is unwanted, non-toxic synthetic hydrogels such as PEG and PHEMA are viable options. However, it has been demonstrated that PEG can trigger foreign body response and although non-biodegradable it can be damaged by acids, reactive oxygen intermediates, enzymes, *etc.*, discharged by macrophages and foreign body giant cells (Browning et al., 2014). Zwitterionic hydrogels have recently been introduced as an excellent antifouling and non-degradable material. Owing to their superhydrophilicity, their stability *in vivo* has for instance been proven for up to 1 year in mice (Dong et al., 2021).

### 3.7 Other uses

Finally, beyond microfabricated bioelectronic interfaces *per se*, hydrogels have found employment as structural materials in related applications. Saeki et al. (2020) have reported on the use of alginate as sacrificial matrix to fabricate protein-based microfibers (Li S. et al., 2020). Another scope of particular interest is the fabrication of scaffolds for artificial organs, enabling biomimetic replicas of biological structures for *in vitro* testing prior to implantation, contributing to a reduction in the use of animals for experimentation (Tringides et al., 2021).

In sum, extensive research has been published to date on the synthesis, functionalization and characterization of hydrogels. However, the integration of this class of materials into manufacturing processes for complete microfabricated devices remains today at a seminal stage. The following section introduces the methods that have been used by researchers to integrate hydrogels into the process flow for microfabricated bioelectronic devices, and presents an understanding of the status of the technology as well as future challenges.

## 4 Integration of hydrogels in microfabricated bioelectronic devices

**FIGURE 2:**
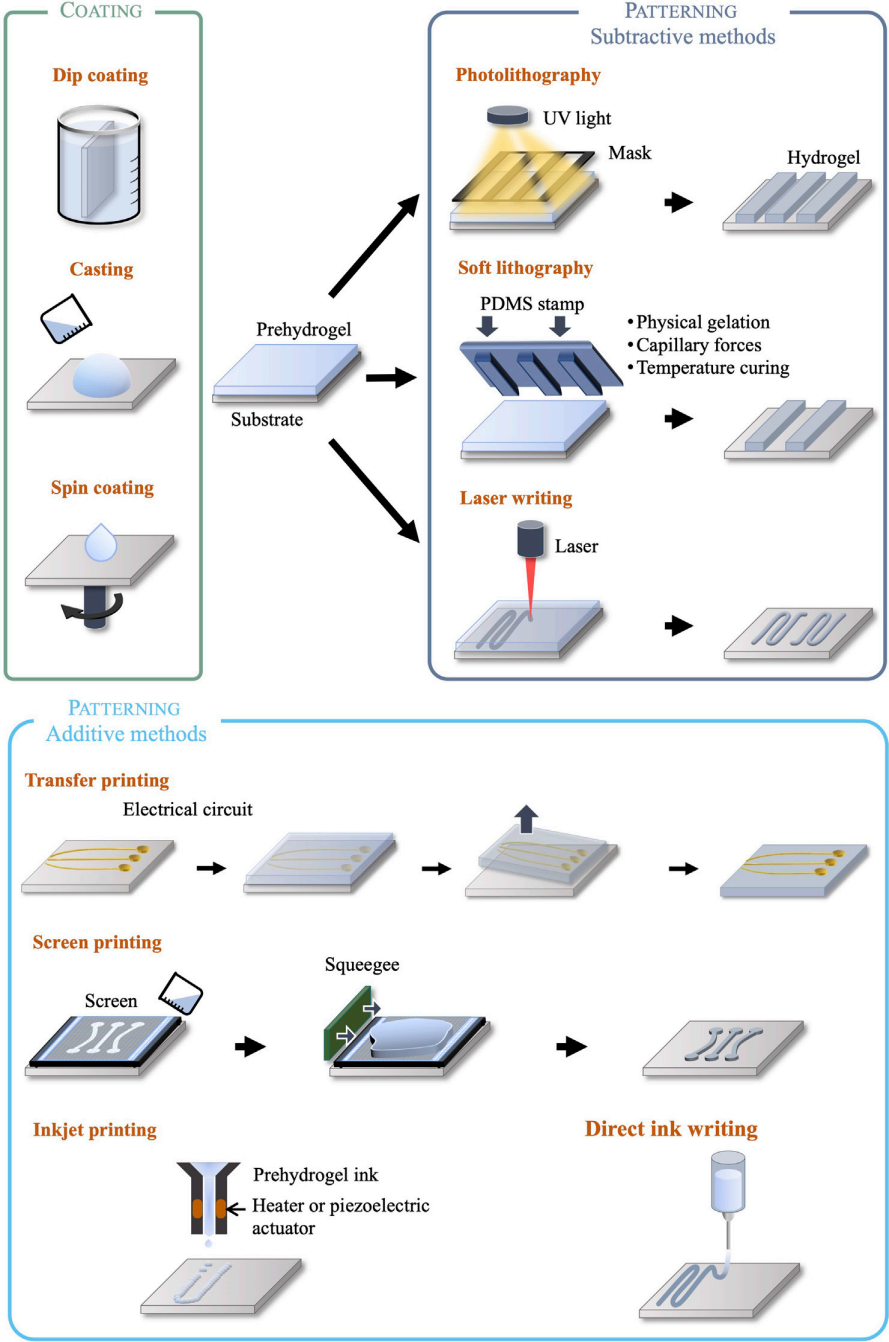
Diagram of the possible microfabrication techniques to deposit and pattern hydrogels.

We classify microfabricated bioelectronic devices with integrated hydrogel layers into two different categories: devices using hydrogels as encapsulation only, to form the chemo-mechanical interface with biological tissue; and devices using hydrogels as elements performing engineered functions (electrical, drug release, *etc.*). This classification based on device architecture is matched by a corresponding classification of the associated manufacturing methods. Typically, encapsulating devices with a hydrogel layer or shell is achieved using non-selective coating processes such as dip coating, drop casting or spin coating. When, however, devices integrate functional hydrogel elements, further micropatterning processes are required in addition to the previous coating processes, introducing a higher level of manufacturing complexity. To this end, conventional silicon and MEMS foundry techniques are not suited for soft and wet materials, and manufacturing processes must therefore be adapted or developed anew by avoiding, for instance, high temperatures and incompatible chemicals. Ad-hoc microfabrication methods for the patterning of hydrogel structures include both subtractive techniques, such as photo- and soft lithography and laser patterning, and additive techniques such as inkjet, direct ink, and screen printing. These are illustrated in Figure 2 and discussed in the following sections.

### 4.1 Coating techniques

Bioelectronic devices that use hydrogels as substrate and/or encapsulation can be fabricated using drop casting and spin-coating methods to deposit the gel on a flat surface such as a glass or silicon carrier (Macron et al., 2019; Zhou et al., 2020), or dip coating of arbitrarily-shaped surfaces (Yin et al., 2018). An alternative technique to coat hydrogels on organic or inorganic surfaces is initiated chemical vapor deposition (iCVD), where monomer, initiator and crosslinker are introduced in vapour phase in a vacuum chamber. A heated filament activates the initiator and hydrogel synthesis occurs on the substrate surface kept at room temperature. High precision thickness and topography can be achieved without subjecting the substrate to high temperatures or solvents (Yagüe and Gleason, 2012).

### 4.2 Photolithography

Photolithography is one of the most common patterning techniques, where a hydrogel precursor solution (prehydrogel) previously coated on a carrier surface is illuminated through a photomask. The light patterns generated by the mask lead to selective crosslinking or polymerization of the illuminated areas, while leaving the rest of the material soluble to a developing agent. This well-established technique offers several advantages such as repeatability, infrastructure availability, the ability to form high resolution patterns (down to 100 nm, and 50 nm using deep UV) (Xu and Siedlecki, 2017), arbitrarily complex in-plane shapes, and multilayer structures by sequential lithographic steps (Tenje et al., 2020). However, the use of photolithography is limited to photosensitive crosslinkers or initiators that are usually cytotoxic due to the radical molecules needed to induce the chemical reactions (Fedorovich et al., 2009; Mironi-Harpaz et al., 2012). In some specific cases of prehydrogels loaded with cells or proteins, UV exposure is of concern for cell viability (Masuma et al., 2013). Lastly, it has been reported that the opacity of photolithographically synthesized hydrogel can cause uneven light exposure, leading to crosslinking gradients within the matrix (Tenje et al., 2020).

### 4.3 Soft lithography

Soft lithography refers to the fabrication or replication of patterns using elastomeric stamps or moulds, commonly PDMS, enabling resolutions down to 100 nm. Patterned stamps are placed in contact with the precursor solution, and capillary forces, heat, physical gelation, chemical or UV crosslinking/polymerization form hydrogel patterns matching the stamp. Contrary to photolithography, the resolution is not limited by optical diffraction, but wettability, Van Der Walls and capillary forces (Nur and Willander, 2020). The main benefits of soft lithography are high resolution and low cost for mass production, suitability to biological samples, compatibility with a wide variety of materials, regardless of their photosensitivity. However, moulds and stamps are manufactured using photo- or e-beam lithography (for nanostructures), which may reduce the cost benefits. For precursor hydrogel solutions requiring UV light to induce polymerization, photocrosslinkers can be detrimental to cells and proteins. Multilayer devices have not yet been demonstrated with soft lithography due to alignment challenges (Nur and Willander, 2020).

### 4.4 Laser patterning

Laser patterning refers to additive and subtractive methods of shaping hydrogels. Direct laser patterning exploits a highly focused light beam to induce localized polymerization or crosslinking to create complex 3D structures. Stereolithography and two-photon photopolymerization are the most reported techniques using lasers. In stereolithography, the precursor hydrogel solution is placed in a tank where the hydrogel is shaped layer by layer through light exposure according to a predefined pattern. The thickness of each layer of the final hydrogel is determined by the motion of a vertical stage. Two-photon polymerization uses femtosecond pulsed lasers to locally crosslink the precursor solution placed in a reservoir in a spatially controlled manner. Similarly, to photolithography, these methods are limited to photosensitive hydrogels precursors. However, it is worth mentioning that hydrogels loaded with cells or proteins would benefit from two-photon polymerization process, as this technique uses near IR which is less harmful for living organisms than UV light (Tenje et al., 2020).

Laser cutting is the subtractive alternative to laser polymerization, where the laser is used to locally break bonds and shape hydrogel structures. It is noteworthy that the opacity of the hydrogel limits the penetration depth of light. Both techniques can achieve µm resolution, but are time consuming (serial process) and require *ad hoc* tools (Verhulsel et al., 2014).

### 4.5 Inkjet and direct ink writing

Inkjet printing is technique in which hydrogel drops are dispensed at precise locations to form predefined patterns. A heater or piezoelectric actuator is used to eject droplets with resolution of 50–500 μm, at speeds up to 5000 drops/s. For hydrogels, the process requires rapid crosslinking and is constrained by the viscosity of the ink (1–15 mPa s in the case of thermal actuators and up to 100 mPa s for piezoelectric actuators), so as to avoid nozzle clogging (Yanagawa et al., 2016; Makrygianni et al., 2018).

Building on inkjet printing, direct ink writing is an additive method enabling the fabrication of complex three-dimensional structures. A viscous hydrogel precursor solution is extruded through a nozzle using pneumatic or screw actuation, and 3D structures are built layer-by-layer on a stage by temperature solidification, physical or chemical gelation. Viscosity, gelation kinetics, sheer-thinning and thixotropic properties are crucial parameters for process development (Liu S. et al., 2020). Direct ink writing has been demonstrated with collagen (Kim et al., 2016), gelatin (Billiet et al., 2014), chitosan (Wu et al., 2018) and alginate (Li et al., 2017). Resolutions down to 30 µm can be achieved (Yuk et al., 2020).

For both techniques, it is possible to integrate biological elements to the ink (e.g., proteins), notably to replicate extracellular matrix and favour cellular growth and differentiation (Nam and Park, 2018). In this case, this method is referred to as bioprinting.

### 4.6 Transfer printing

Transfer printing enables electrical circuits fabricated on a separate donor substrate using conventional methods to be transferred onto a hydrogel acceptor substrate. The electrical conductors are first patterned on the donor substrate. Next, a hydrogel layer is deposited on top of the patterned conductors and then lifted off. This method eliminates the need for the hydrogel to be suitable as substrate for subsequent conductor deposition and patterning. However, the technique relies on the careful interplay of adhesion forces between donor, transfer patterns, and the hydrogel substrate (Zhou et al., 2019).

### 4.7 Screen printing

Screen printing enables the formation of patterns on a surface by applying a viscous material through a screen and a stencil mask, which is machined to match the desired patterns and aligned to the underlying substrate. This technique offers the advantages of low cost and ease of manufacturing, a resolution down to 300 µm (He et al., 2019; Pandala et al., 2020) and sterilized stencils can be used to fabricate cell culture hydrogel scaffolds (Pandala et al., 2021).

Despite the wide range of possible techniques made available from the microelectronic manufacturing industry, their application to hydrogel materials as part of complete bioelectronic devices has not yet benefitted from collective efforts in standardization and adoption. The technological processes used today to fabricate the devices presented in the scientific literature are inherited from silicon or MEMS foundry, and adapted case-by-case to specific materials and designs. Wide applicability of these techniques has therefore not yet been achieved.

## 5 Applications in bioelectronic medicine and technology readiness levels

**FIGURE 3:**
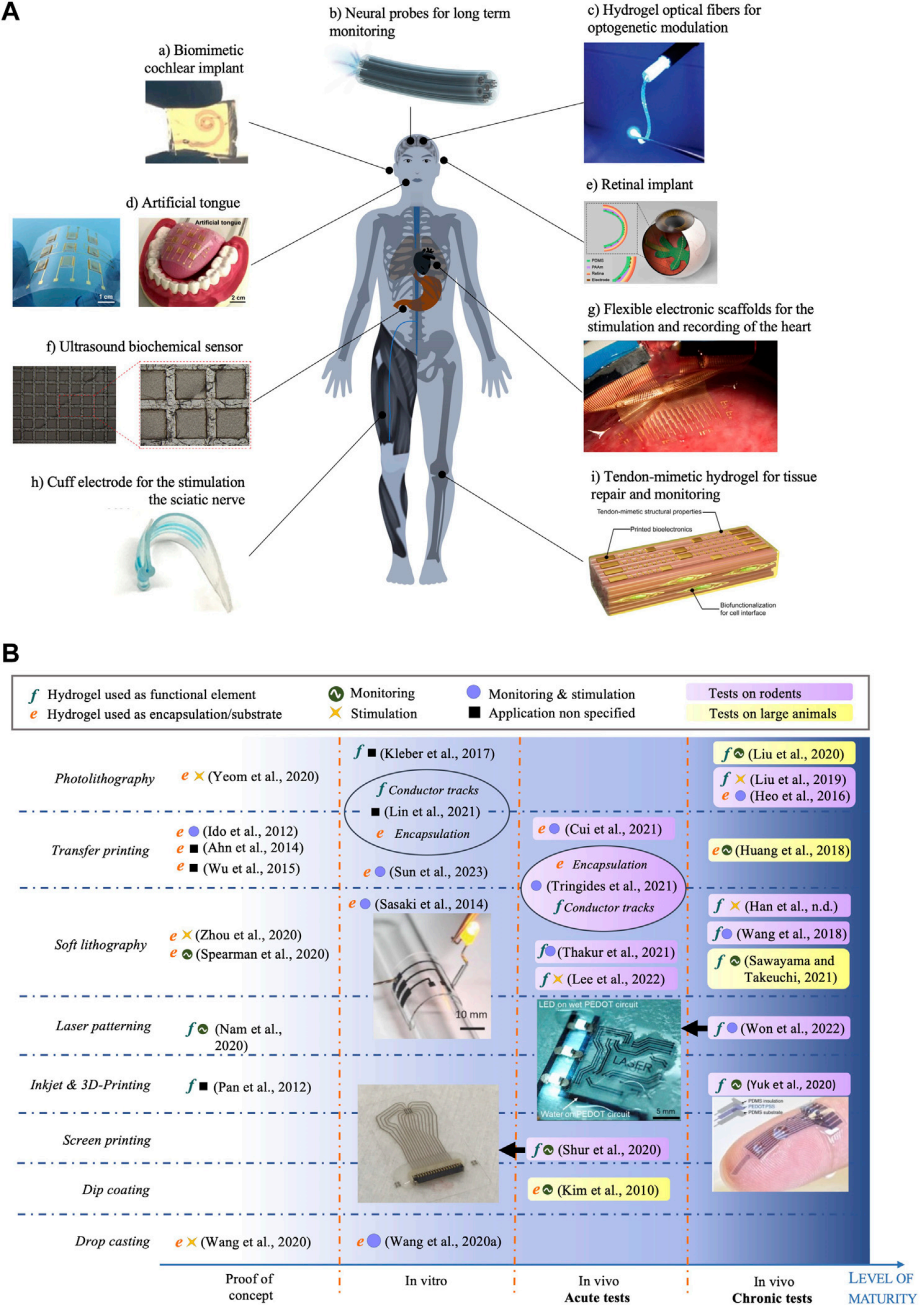
**(A)** Example applications of hydrogel-based bioelectronic devices. Reproduced with permission from reference: (a) (Lei et al., 2017), copyright 2021 Springer Nature. (b) (Park et al., 2019), copyright 2020 Springer Nature. (c) (Wang et al., 2017), copyright 2018 Wiley-VCH. (d) (Yeom et al., 2020), copyright 2020 American Academy for the Advancement of Science. (E) (Zhou et al., 2020). (f) (Nam and Park, 2018), copyright 2020 Frontiers. (g) (Li J. et al., 2020). (h) (Thakur et al., 2021). (i) (Sun et al., 2023), copyright 2023 American Academy for the Advancement of Science. **(B)** Map of hydrogel-based bioelectronic devices according to the microfabrication technique to process hydrogel layers (rows) and their level of maturity (x-axis). Reproduced with permission from reference (Sasaki et al., 2014), (Won et al., 2022), (Yuk et al., 2020) and (Shur et al., 2020). Copyright 2014 Wiley, 2022 American Academy for the Advancement of Science, 2020 Springer Nature and 2020 American Chemical Society respectively.

Due to their versatility in functionalisation, shape and stimuli-responsiveness, hydrogels have been extensively explored for medical applications: from contact lenses, dentistry (Han et al., 2017), surgical adhesives (Zhang et al., 2020), cartilage treatment (Chuang et al., 2018; Wei et al., 2021), bone regeneration (Kuroda et al., 2019; Lee C.-S. et al., 2020), to drug delivery (Raina et al., 2022). Several hydrogel materials developed for tissue engineering are currently being tested in clinical trials or have been granted authorization for commercialization (Mandal et al., 2020). However, microfabricated bioelectronic devices using hydrogels either as functional electrical elements or as passive interface layers are still in their infancy, with research still at an early stage, as shown by the range of publication dates. While many proofs of concept in the literature provide elements of feasibility and relevance, a clinic-ready, let alone commercially available, bioelectronic device, is yet to be found. Figure 3A displays a selection of hydrogel-based bioelectronic devices reported in the scientific literature, with illustrations of their intended deployment. Table 2 lists a selection of notable examples of complete bioelectronic devices, classified according to the microfabrication process employed, and scored on a qualitative scale indicating the level of maturity based on the preclinical validation data reported.

**TABLE 2:** Selection of hydrogel-based bioelectronic devices, classified according to the fabrication technique. Devices are qualitatively scored based on a level of maturity (LM) scale: 1- Proof of concept (mechanical, electrical and electrochemical testing); 2- *In vitro* testing (e.g., cytotoxicity); 3- *In vivo* acute testing on small animals (rodents); 4- *In vivo* acute testing on large animals (pigs, monkeys, cats, dogs); 5- *In vivo* chronic testing on rodents; 6- *In vivo* chronic testing on large animals; 7- Clinical trials.

Fabrication process	Hydrogel material	Function of the hydrogel in the device	Application	LM	Key metrics	Resolution	Ref
Photo-lithography	PEDOT: PSS hydrogel	Conductive hydrogel encapsulated in elastomer PFPE-DMA	Stimulation of the sciatic nerve	5	• σ = 47.4 ± 1.2 S/cm	2 µm	Liu et al. (2019)
					• t_H_ = 200 nm		
					• E = 32 ± 5.1 kPa (compression)		
					• Z > 100 MΩ		
	PEG	Coating of the electrode, hydrogel loaded with anti-inflammatory drugs and PEDOT:PSS	Stimulation of the sciatic nerve	1	• Z = 580.2 ± 40.1 Ω (1 kHz)	200 µm	Heo et al. (2016)
					• t_H_ = 208 ± 11 μm		
					• CDC = 2.67 ± 0.37 μC/mm^2^		
Soft lithography	HA–Collagen I–laminin	Encapsulation of polyimide microelectrodes	TE electronic nerve interface	1	• Diameter = 1mm	1 mm	Spearman et al. (2020)
					• E_H_ = 2.55 ± 0.05 kPa		
	PU	PU encapsulation and PEDOT/PU–hydrogel hybrid (working electrode)	Advanced tissue engineering including electronics	2	• σ = 120 S/cm	40 µm	Sasaki et al. (2014)
					• t_H_ = 200µm		
					• Elongation ratio = 100%		
Direct ink writing and Inkjet printing	PEDOT:PSS hydrogel	PEDOT: PSS electrical circuit encapsulated in PDMS	Neural probe	5	• E = 1.1 MPa	30 μm	Yuk et al. (2020)
					• σ = 28 S/cm		
					• Z = 50–150 Ω (1 kHz)		
	PANI hydrogel	Conductor track	N.A.	1	• Capacitance = 480 F/g	18 μm	Pan et al. (2012)
					• R = 3.2 Ω		
					• σ = 0.23 S/cm		
Transfer printing	Fe^3+^ -[PEG-Dopa]_4_	Encapsulation	Microelectrode array for recording the sciatic nerve	6	• E = 17.9 ± 0.3 kPa	25 μm	Huang et al. (2018)
					• t_H_ = 1 mm		
					• Z = 32.2 ± 8.3 kΩ (f = 1 kHz)		
	Gelatin and GelMa	All hydrogel-based device GelMa doped with Ag NWs, Pt NWs, and PEDOT:PSS	Microelectrode array for neural interface	2	• Z_GelMa_ = 38.3–52.4 Ω (f = 1 kHz)	30 µm	Lin et al. (2021)
					• E = 180 kPa		
					• R_s_ = 592 ± 22.7 Ω		
Laser patterning	PEDOT: PSS	PEDOT: PSS hydrogel encapsulated in PDMS	Stimulation and recording of the sciatic neve	5	• E = 57 MPa	6 µm	Won et al. (2022)
					• σ = 670 S/cm		
					• Z = 6 kΩ (f = 1 kHz)		

Figure 3B provides an overview on the status of the technology, with a map of all complete hydrogel-based microfabricated bioelectronic devices published in the literature, to the best of our knowledge. We classify the devices according to the technique used to process the hydrogel material (rows) and the level of maturity based on the validation data presented (x-axis). This map reveals that most of the published work includes acute and chronic tests conducted with rodents to confirm the ability to deliver electrical stimulation or to record electrophysiological responses. In smaller proportion, device operation tests with pigs (Sawayama and Takeuchi, 2021), cats (Huang et al., 2018) and rabbits (Xue et al., 2021) have been reported. In terms of microfabrication approach, the majority of the reported devices are manufactured using photolithography, soft lithography and transfer printing techniques, owing to the wider availability of the associated equipment. Some of the devices integrate hydrogels both as a functional electrical element and as an encapsulation using different microfabrication methods (Lin et al., 2021; Tringides et al., 2021). Finally, we note that no device has been tested in clinical trials yet, testifying to the novelty of this material technology. Some devices were tested chronically *in vivo* on both rodents and large animal models, suggesting a translational pathway planning. The longest *in vivo* implantation period reported thus far is 6 weeks. For example, Liu et al. (2020a) successfully implanted a hydrogel based electrode for chronic epicardial and endocardial mapping of the heart in a pig for 6 weeks. Won et al. (2022) showed that electrodes made of PEDOT:PSS encapsulated in soft SBS produced minimal damage to the sciatic nerve tissue in comparison with a rigid Au cuff electrode control, after 4 weeks implantation in mice.

## 6 Discussion

Hydrogels have extensively gained research interest over the past two decades (from less than 500 publications per year in 2002 to over 10,000 in 2022) (Correa et al., 2021). Owing to their wide range of property tunability, they are promising interface materials for mimicking the mechanical, chemical and electrical properties of biological tissues. Most contributions to the scientific literature focus on the synthesis and structuring of hydrogels, both at the micro and macro scale. However, full integration of hydrogel materials into minimally invasive and biomimetic bioelectronic devices is yet to be achieved at large scale, as numerous important technological and manufacturability challenges remain unsolved.

Patterning small features on hydrogels for long term implantation is a challenging endeavour, as they tend to degrade much faster compared to inorganic materials. Biostability data varies widely: Wang et al. (2017) reported that 87% of a chitosan/alginate hydrogel degraded after 12 days *in vivo*, while Browning et al. (2014) showed PEGDA hydrogels, subcutaneously implanted, stabile for up to 12 weeks. Stability in biological media over time is an essential requirement that has yet to be widely demonstrated with hydrogel-based devices. A possible strategy to address this challenge is to use synthetic monomers to further increase the lifetime of the structure (Takeda et al., 2015; Loh et al., 2020). In general, however, beside isolated examples, developing robust and standardized manufacturing methods accessible to the wider bioelectronic microfabrication community has yet to be achieved to unlock reproducible manufacturability of hydrogels at the microscale.

Another consideration that is specific to bioelectronic interfaces, is the unsuitability of hydrogels as barrier layers encapsulating electrical conductors. While the advantages of soft materials in reducing the mechanical mismatch to biological tissue are well-established, complete and stable bioelectronic devices cannot be made entirely of hydrogels, as these cannot guarantee the necessary insulation when biological fluids are absorbed. Given enough diffusion time, their intrinsic permeability to ionic fluids renders the entire volume conductive, bridging conductors intended to carry separate electrical signals. To overcome this challenge, elastomers with a slightly higher modulus (MPa range) have been proposed to encapsulate and electrically insulate the conductors, while containing the consequent decrease in mechanical compliance (Liu et al., 2019). Hydrogels can also be coated on thin elastomer substrates to provide the interface with the biological tissue at specific locations (Tringides et al., 2021). This strategy trades off elasticity with acceptable barrier performance. In general, although considerable advances have been reported in building interfaces that mimic biological tissue, the path to a fully synergistic bioelectronic device remains yet unpaved.

The limitations above, coupled to other specific design challenges such as the management of swelling and the associated interfacial stresses, constitute a significant set of roadblocks that today hinders the surfacing of complete hydrogel-based bioelectronic devices ready for clinical use or long-term preclinical validation. The authors expect a near-future increase in the research momentum to address the challenges presented herein, with the aim of enabling microelectronic manufacturing of fully biomimetic bioelectronic interfaces.

## Glossary

2PP: Two-photon polymerization
AG: Arabic gum
Anti-HBc: Hepatitis B core antibody
CDC: Charge delivery capacity (charge capacity per unit area)
CNC: Cellulose nanocrystal
DA: Dimethylacrylate
DE: Diglycidyl ether
Dopa: Dopamine
E: Young’s modulus
GelMa: Gelatin methacryloyl
GMA: Glycidyl methacrylate
IPN: Interpenetrating polymer network
LOx: Lysyl oxidase
NWs: Nanowires
PAA: Polyacrylamide
PAAN: Polysodium acrylate
PACP: Poly(aniline-co-pyrrole)
PAMAM: Poly(amido amine)
PANi: Polyaniline
PBMA: Poly(butyl methacrylate)
PCL: Polycaprolactone
PDCA: Pyridinedicarboxamide
PDMS: Polydimethylsiloxane
PEDOT: Poly(3,4-ethylenedioxythiophene)
PEG: Polyethylene glycol
PEG-SVA: Poly(ethylene glycol disuccinimidyl valerate)
PEG-VS: Poly(ethylene glycol) vinyl sulfone
PFPE-DMA: Dimethacrylate-functionalized perfluoropolyether
PGA: Poly(glycolic acid)
PHEMA: Poly(hydroxyethyl methacrylate)
PI: Polyimide
PMEP-DMA: Poly(methyl ethylene phosphate)-dimethacrylate
PMMA: Poly(methacrylic acid)
PNH: Poly(N-isopropylacrylamide-co-2-hydroxyethyl methacrylate)
PPy: Polypyrrole
PU: Polyurethane
PSS: Polystyrene sulfonate
PVA: Polyvinyl alcohol
R: Resistance
**R**_**s**_: Sheet resistance
SBS: Styrene-butadiene-styrene
SU-8: Structured by UV (refers to the epoxy-based negative photoresist)
**t**_**H**_: Thickness of the hydrogel layer
Z: Impedance
σ: Electrical conductivity
